# Seasonal metabolic flexibility is correlated with microclimate variation in horned larks and house sparrows

**DOI:** 10.1093/cz/zoab037

**Published:** 2021-05-04

**Authors:** Paige Oboikovitz, David L Swanson

**Affiliations:** Department of Biology, University of South Dakota, Vermillion, SD 57069, USA; Department of Biology, University of South Dakota, Vermillion, SD 57069, USA

**Keywords:** climatic variability hypothesis, *Eremophila alpestris*, metabolic flexibility, metabolic rates, microclimates, *Passer domesticus*

## Abstract

Maximum and minimum metabolic rates in birds are flexible traits and such flexibility can be advantageous in variable climates. The climatic variability hypothesis (CVH) posits that more variable climates should result in greater metabolic flexibility for geographically distinct populations. Whether the CVH applies to sympatric species occupying microclimates differing in variability is unknown. Microclimates of open habitats are likely more variable than those of sheltered habitats. If the CVH extends to microclimates, we expect birds from open habitats to show greater flexibility than those from sheltered habitats. To test this extension of the CVH, we compared seasonal variation in microclimates and metabolic rates for sympatric horned larks *Eremophila alpestris*, which occupy open habitats, and house sparrows *Passer domesticus*, which occupy sheltered habitats. We measured operative temperature (*T*_e_, an integrative measure of the thermal environment), summit metabolic rate (*M*_sum_, maximal cold-induced metabolic rate), and basal metabolic rate (BMR, minimal maintenance metabolic rate) in summer and winter. For both winter and summer, daily minimum *T*_e_ was similar between open and sheltered habitats but maximum *T*_e_ was higher for open habitats. Winter microclimates, however, were colder for open than for sheltered habitats after accounting for convective differences. Both species increased *M*_sum_ in winter, but seasonal *M*_sum_ flexibility was greater for larks (43%) than for sparrows (31%). Winter increases in BMR were 92.5% and 11% for larks and sparrows, respectively, with only the former attaining statistical significance. Moreover, species * season interactions in general linear models for whole-organism metabolic rates were significant for BMR and showed a similar, although not significant, pattern for *M*_sum_, with greater seasonal metabolic flexibility in horned larks than in house sparrows. These results suggest that extending the CVH to sympatric bird species occupying different microclimates may be valid.

Small birds in temperate regions are faced with marked seasonal changes in temperature to which they respond by reversibly altering metabolic phenotypes to better match environmental conditions (Swanson 2010), a process known as phenotypic flexibility (Piersma and Drent 2003). As a result, small birds from cold winter climates typically elevate basal (BMR) and summit (*M*_sum_, maximum thermogenic capacity) metabolic rates and tolerate exposure to colder temperatures in winter relative to summer (Marsh and Dawson 1989; Swanson 2010). Increasing metabolic rates to high levels and sustaining these levels over an extended period of time is energetically expensive and involves various physiological and biochemical changes (Swanson 2010). Adjusting metabolic rates to match environmental demands allows upregulation when high metabolic rates are required and downregulation to avoid excess energetic costs when they are not. Such metabolic flexibility can have fitness consequences (Nilsson and Nilsson 2016; Petit et al. 2017; Latimer et al. 2018). Nevertheless, there are physiological limits to elevating metabolic capacity, although the precise mechanistic causes of these limits are still poorly understood (Lundby and Robach 2015). *M*_sum_ varies within and among species, as well as geographically and seasonally (Swanson 2010; Swanson and Vézina 2015). For example, house finches (*Haemorhous mexicanus*) from the cooler climates of Colorado had higher *M*_sum_ than those from California in both summer and winter, suggesting that regional climates influenced metabolic capacity (Dawson et al. 1983). Moreover, house finches from Michigan and South Dakota also had higher winter *M*_sum_ than California birds, although summer *M*_sum_ among these populations were similar (O’Connor 1995; Swanson and Liknes 2006). Winter BMR and *M*_sum_ are consistently greater than summer metabolic rates for most small birds in cold winter climates (Liknes et al. 2002; Swanson 2010; Petit et al. 2013), but not consistently for birds from subtropical or tropical climates (Wells and Schaeffer 2012; McKechnie et al. 2015; Noakes et al. 2020). *M*_sum_ and BMR may also differ from winter to winter, with colder winters producing higher metabolic rates (Swanson and Olmstead 1999; Petit et al. 2013).

Flexible metabolic phenotypes are advantageous when the benefits of the response to short-term environmental changes are greater than the long-term costs associated with maintaining a flexible phenotype, so a strong relationship between flexibility and environmental variability would be expected (Via and Lande 1985; DeWitt et al. 1998; Piersma and Drent 2003). Such a relationship between phenotypic flexibility and environmental variation is proposed by the climatic variability hypothesis (CVH), which posits that geographically separated populations inhabiting variable environments should exhibit greater physiological flexibility than populations from stable environments (Gaston and Chown 1999; Cavieres and Sabat 2008). Several studies have attempted to test the CVH with reference to metabolic flexibility in birds. For example, Cavieres and Sabat (2008) found a positive relationship between flexibility of BMR and climate variability in rufous-collared sparrows *Zonotrichia capensis*. Similarly, *M*_sum_ flexibility was positively correlated with environmental temperature variability in junco (*Junco hyemalis* and *J. phaeonotus*) populations (Stager and Senner 2020). In contrast, several studies failed to find significant relationships between flexibility in BMR or *M*_sum_ and climate variability (Tieleman et al. 2003; van de Ven et al. 2013; Noakes et al. 2017; Noakes and McKechnie 2020). Thus, although some support for the CVH exists for birds, it does not appear to apply uniformly across different locations with different magnitudes of climatic variation. In addition, Swanson et al. (2020) found that metabolic flexibility was not associated with within-season temperature variability in house sparrows *Passer domesticus*, suggesting that the CVH does not apply at the within-population, seasonal scale in birds.

Physiological and biochemical adjustments associated with metabolic flexibility can be energetically expensive, so to reduce energetic costs associated with thermoregulation, birds also show behavioral adjustments, such as seeking favorable microclimates (Petit and Vézina 2014). Heterogeneous environments produce a suite of microclimates that can influence energetic demands for species occupying such environments. Whether the CVH also applies to sympatric species occupying microclimates differing in temperature variability is unknown. Microclimates of open, sparsely vegetated habitats are more exposed and likely more variable than those of more sheltered habitats. If the CVH extends to microclimatic variation in regions of sympatry, we would expect birds from open habitats to show greater metabolic flexibility than those from more sheltered habitats. In support of this idea, black-capped chickadees *Poecile atricapillus* from landscapes with smaller and more fragmented woodlands, with greater wind penetration, had higher *M*_sum_ and body mass than other populations (Olson et al. 2010), potentially as a response to thermal challenges associated with higher convective heat loss (Dolby and Grubb 1999; Olson and Grubb 2007).

The reduction in metabolic costs when using a suitable microclimate can vary with the structural features of the environment. For example, verdins *Auriparus flaviceps*, moving from a shady, windy site to a sheltered, sunny site could reduce metabolic rates for thermoregulation by 50% (Wolf and Walsberg 1996). Similar patterns of reduced metabolic costs are evident for roost site selection in overwintering birds. Choosing roost sites in dense branches of spruce trees significantly reduced radiative and convective heat exchanges with the external environment for American goldfinches *Spinus tristis*, which enabled them to endure cold overnight temperatures as low as −40°C (Buttemer 1985). Cavity roosting for wintering juniper titmice *Baeolophus griseus* and mountain chickadees *Poecile gambeli* reduced energy expenditure by 25.1–37.6%, respectively, as opposed to roosting outside cavities (Cooper 1999). Ruby-crowned kinglets *Regulus calendula*, one of the smallest passerine birds, may occupy sheltered roost sites in winter (Farley 1993; Strauss and Swanson 2020) and such sites could reduce energetic costs relative to roosting in open sites by at least 18%.

The objective of this study was to test whether the CVH may be extended to sympatric bird species occupying different microclimates. Specifically, we explored the relationship between temperature variability of microclimates and metabolic flexibility by comparing the seasonal thermal microclimates, measured as *T*_e_ (an integrative measure of the thermal environment to which and animal is exposed; Bakken and Gates 1975; Bakken 1976; Bakken et al. 1985), and summit and BMR of house sparrows and horned larks in southeastern South Dakota. These 2 species are permanent residents in South Dakota (Tallman et al. 2002) but occupy habitats differing in structural heterogeneity. House sparrows are generally found in wooded and human-developed areas (Lowther and Cink 2006) whereas horned larks prefer bare, dry ground, and areas of sparse vegetation (Beason 1995). More sheltered and shaded microclimates created by buildings or woodlands that are occupied by house sparrows, including nesting or roosting in cavities in trees or on buildings (Lowther and Cink 2006), can provide more stable thermal conditions because they reduce exposure to heat and wind. In contrast, open habitats like prairies, grasslands, and agricultural fields occupied by horned larks are more exposed to weather conditions due to fewer structural features providing relief from solar radiation and wind. Moreover, other lark species from open habitats appear to have substantial capacity for metabolic flexibility (Tieleman et al. 2003). Because of the difference in structural heterogeneity of habitats occupied by these 2 species, we hypothesized that there will be greater seasonal variation in *T*_e_ in the open field sites occupied by horned larks than in the more protected sites occupied by house sparrows. We also hypothesized that horned larks will have lower metabolic rates in summer and higher metabolic rates in winter than house sparrows because they experience higher operative temperatures in summer and lower operative temperatures in winter, and therefore, should alter their metabolism to a greater magnitude on a seasonal basis. Thus, consistent with the CVH applied to microclimatic variation, we predict that the seasonal differences in both summit and BMR will be greater for horned larks than for house sparrows because of greater seasonal variability in the microclimates occupied by horned larks.

## Materials and Methods

### Bird capture

We captured house sparrows (Winter: *n = *19; Summer: *n = *12) and horned larks (Winter: *n = *3; Summer: *n = *4) before 11:00 CST using mist nets near Vermillion, Clay County, South Dakota (42.7794°N, 96.9292°W) during winters (December–February) and summers (June–August) of 2016 and 2017. All birds used for the measurements were adults, with the exception of 1 juvenile horned lark in summer, for which body mass and metabolic rate values fell within the range of adult birds. We used feeders, playbacks, and carved decoys (for horned larks only) to lure birds to the nets. After a bird was captured, we transported it directly to the laboratory within 10–20 min. In the laboratory, we housed birds in small flight cages with ad libitum mixed bird seed and water both before and after metabolic measurements, which took place on the day of capture. After metabolic trials, we attached a standard USFWS aluminum leg band and released birds the following morning at the site of capture. Winter horned lark sample sizes for metabolic rate measurements were supplemented with data from a previous study (*M*_sum_, *n = *4; BMR, *n = *2), as these birds were collected from the same study area and metabolic measurements were conducted using identical methods (Swanson and Liknes 2006, unpublished data). We typically measured both *M*_sum_ and BMR for most birds. However, because we could only measure BMR on a maximum of 2 birds per night, we lack some BMR measurements on birds for which we measured *M*_sum_. In addition, we measured BMR for 1 summer-collected house sparrow for which we did not measure *M*_sum_. Thus, total sample sizes for metabolic rate measurements were: house sparrow summer, *M*_sum_  *n = *12, BMR *n = *13; house sparrow winter, *M*_sum_  *n = *19, BMR *n = *17; horned lark summer, *M*_sum_  *n = *4, BMR *n = *3; horned lark winter, *M*_sum_  *n = *7, BMR *n = *4. All procedures in the study were approved by the Institutional Animal Care and Use Committee at the University of South Dakota (Protocol Number 19-11-14-17C) and capture occurred under federal (MB-758442-0) and state (15-6,16-8 and 17-8) scientific collecting permits.

### Summit and BMR measurements

We measured *M*_sum_, typically beginning a few hours after the birds were brought into the lab, from ∼10:00 to 14:00 CST. We measured *M*_sum_ by open-circuit respirometry using a 1.9 L steel metabolic chamber with its interior painted black to ensure that metabolic heat produced by the bird was transferred out of the chamber. To prevent cold-induced injuries and to achieve measures of summit metabolic rates at modest sub-freezing temperatures, we used a helium–oxygen (helox) gas mixture (79% helium/21% oxygen) for the respiratory gas (Rosenmann and Morrison 1974; Holloway and Geiser 2001; Arens and Cooper 2005). We controlled temperature of the metabolic chamber by submerging it into a bath of ethylene glycol and water to achieve sub-freezing temperatures. Because winter birds are more cold tolerant than summer birds (Swanson and Liknes 2006), the starting temperature for *M*_sum_ trials was −9°C in winter and 0°C in summer. This allowed the induction of hypothermia, which marked the end of cold exposure tests, to occur over similar time periods (generally 45–90 min, except for horned larks in winter which tolerated cold for longer periods) in both summer and winter. We recorded baseline measures of oxygen concentration in incurrent gas before and after the metabolic trial. After we placed the bird inside the metabolic chamber, we flushed the chamber with helox for 5 min to allow equilibration. We then submerged the chamber into the ethylene glycol bath. We measured oxygen concentration in the excurrent gas flowing out of the chamber every 2 s with an Ametek S-3AII Oxygen Analyzer (Ametek Process Instruments, Berwyn, PA) and recorded data with ExpeData version 2.0 (Sable Systems, North Las Vegas, NV). Each day prior to metabolic measurements, we calibrated the oxygen analyzer with room air and helox (*M*_sum_ only), which were scrubbed of carbon dioxide (CO_2_) and water before and after the metabolic chamber using Drierite (Anhydrous Calcium Sulfate; W.A. Hammond Drierite Co., Xenia, OH) and Ascarite (Sodium hydroxide-coated silica; Aldrich Chemical Company, Inc., Milwaukee, WI). We maintained a flow rate of 1,010–1,030 mL min^−1^ during *M*_sum_ recordings with a Cole-Parmer Model Precision Rotameter (Model FM082-03ST; Chicago, IL), which was calibrated to ±1% accuracy with a soap bubble meter.

For *M*_sum_ measurements, we kept the bath at the initial temperature for 20 min and decreased the temperature by ∼3* *°C every 20 min thereafter until the bird exhibited a slow decrease in oxygen consumption indicating hypothermia (Swanson et al. 1996). Once this decline was apparent, the bird was immediately removed from the chamber and cloacal body temperature was measured with a lubricated thermocouple thermometer (Cole-Parmer Model 8500-40; Chicago, IL) and 30-gauge copper-constantan thermocouple, which was inserted into the cloaca to a depth of ∼1 cm. The thermocouple thermometer was calibrated to ±0.3°C accuracy against a mercury thermometer traceable to the US National Institutes of Standards and Technology. Bird body temperatures ≤37°C indicated that hypothermia was reached in all individuals after *M*_sum_ measurements, except for 3 horned larks in winter, which were not hypothermic at the end of the 3-h metabolic trials. We also weighed all birds to the nearest 0.1 g before and after metabolic trials.

We used the same procedure for BMR measurements, except the bath was kept constant at 30°C (a temperature within each species’ thermal neutral zone; Hudson and Kimzey 1966; Trost 1972) and we used dry, CO_2_-free room air as the respiratory gas, at a flow rate of ∼300 mL min^−1^. When 2 birds were caught on the same day, we also used a FoxBox Field Gas Analysis System (Sable Systems International, North Las Vegas, NV) for BMR but not *M*_sum_ measurements. The FoxBox was connected to a second metabolic chamber with the same setup as for the Ametek S-3AII Oxygen Analyzer but linked to a separate computer recording oxygen concentration with ExpeData version 2.0. We calibrated air flow for the FoxBox Mass flowmeter using the same procedure and soap bubble meter as for the rotameter. Both metabolic chambers were submerged into the same bath so that the 2 metabolic trials could be measured simultaneously at identical temperatures. We recorded baselines of oxygen concentrations before and after each BMR measurement. We initiated BMR recordings in the evening (19:30–20:00 local time) but did not remove food prior to BMR trials, allowing birds to become post-absorptive over the duration of the measurement, which lasted overnight (∼12 h). The initiation of BMR measurements occurred at least 6 h after *M*_sum_ measurements for birds on which both metabolic traits were measured. We removed birds from the chamber the following morning (07:30–08:00 CST). We recorded oxygen concentration every 5 s throughout the night.

### Operative temperature measurements

Operative temperatures were recorded during Winter 2016 (6 January to 29 February), Summer 2016 (15 June to 31 August), Winter 2016–2017 (1 December 2016 to 28 February 2017), and Summer 2017 (5 June to 31 August) near Vermillion, Clay County, South Dakota. For each season, we selected 12 locations, 6 in open fields and 6 in sites with woody vegetation surrounding human structures, for the deployment of operative temperature thermometers, except for Winter 2016, which had only 10 locations with 5 open and 5 vegetated sites. All *T*_e_ thermometer locations were sites where we had previously observed either horned larks or house sparrows, so were representative of microclimates occupied by the 2 study species. For house sparrows, these sites consisted of urban and rural areas with woody vegetation around human habitations (Lowther and Cink 2006). For horned larks, these sites consisted of open agricultural fields and grasslands or pastures (Beason 1995). We used the same sites for all seasons and years, with the exception of 3 summer sites in open fields (horned lark microclimates), where floods or agricultural activities destroyed the *T*_e_ thermometers during earlier summer measurements. These 3 *T*_e_ thermometers were deployed in similar open habitats where we had also observed horned larks.

Because our study design involved estimating thermal microclimates of multiple locations simultaneously over a period of 2–3 months, we were not able to use taxidermic mounts of birds for *T*_e_ thermometers. Instead, we estimated *T*_e_ at open and sheltered sites using a modification of the technique described by Walsberg and Weathers (1986) and used previously to describe thermal microclimates of rooftop nest sites of common nighthawks *Chordeiles minor* (Newberry and Swanson 2018). Walsberg and Weathers (1986) compared painted hollow copper spheres with taxidermic mounts to estimate *T*_e_ and found that spheres provided acceptable *T*_e_ thermometers (generally ˂2–4°C differences from taxidermic mounts for hourly measurements, but up to 6.3°C differences for short time scales) for analyses involving multiple measurements over time periods of several hours. We designed operative temperature thermometers from 13 × 10 × 12 cm ovoids (copper toilet floats; Plumb Craft 7644000A) with the outside surface painted flat gray. We recognize that estimates of *T*_e_ from our thermometers may deviate to some extent from those of taxidermic mounts, which may also differ from thermal conditions in living birds (Walsberg and Wolf 1996), but because our study design included many measurements over entire summer and winter periods (up to 3* *months), we contend that our *T*_e_ thermometers do provide a useful estimate of the long-term thermal environment to which birds were exposed.

The *T*_e_ thermometer system consisted of a HOBO UX100-014M Single Channel Thermocouple data logger (Onset Computer Corporation, Bourne, MA) attached to a copper-constantan thermocouple with the tip inserted through a small hole into the center of the copper ovoid. These data loggers record at  ± 0.2°C precision from 0 to 50°C. We calibrated all *T*_e_ thermometer systems at cold (−15 to −20°C), cool (4°C), room (24°C), and hot (50–60°C) temperatures. Temperatures from *T*_e_ thermometers varied from actual temperatures by an average of only ±0.80°C, so we used raw *T*_e_ data for subsequent comparisons. We enclosed the data logger within a water-resistant plastic container and sealed the hole in the copper ovoid with epoxy to make it water tight. We attached copper ovoids in open field sites to wooden dowel rods which were attached to rebar rods that were staked into the ground. This allowed us to place the copper ovoids so that they hung just above the ground. The *T*_e_ thermometers placed in vegetation were secured to branches and the copper ovoids were allowed to hang freely just below the branch to which they were attached. We programmed data loggers to take temperature recordings every 30 s for the duration of the season using HOBOware software (Onset Computer Corporation). Because of battery failure, programming errors, and weather issues causing periods of missed recordings, we had some missing *T*_e_ data for both open and sheltered sites for each season, but these most often covered similar time periods for the 2 sites, except for periods of flooding that compromised 3 dataloggers in open sites in summer. For winter data, we had no missing data for sheltered or open microclimates in January 2016. We did, however, have missing data for sheltered sites for all other winter months, including 3.2% of time points in February 2016, 30.8% in December 2016, 17.3% in January 2017, and 22.9% in February 2017. For winter data from open sites, missing data comprised 9.6% of time points in February 2016, 18.4% in December 2016, 33.9% in January 2017, and 24.8% in February 2017. For sheltered habitats in summer 2016, we had no missing data for June, 3.7% missing data for July, and 14.7% missing data for August (although 1 additional data logger failed to record after late July). Open habitats in summer 2016 had no missing data in June, 1.4% missing data in July, and 9.1% missing data for August (although 1 additional data logger failed to record after late July). During the summer of 2017, we had no missing data for sheltered sites for June or August (although 1 data logger failed to record after 20 July), but July had 22.7% missing data. Open sites in the summer of 2017 had no missing data in June, 31.2% missing data in July, and 0.01% missing data in August, although 2 data loggers failed to record after 20 and 22 July, respectively.

### Standard operative temperature estimation

Because our operative temperature thermometers were not heated models, they do not fully incorporate the effects of wind on thermal conditions to which birds were exposed. To better account for convection effects, we estimated standard operative temperature using an equation derived by Bakken (1990):

$$T_{\text{es}}=T_{b}-\left(1+0.26\surd u\right)\left(T_{b}-T_{e}\right)$$

which converts operative temperature (*T*_e_) into standard operative temperature (*T*_es_) using wind speed (*u*) and body temperature (*T*_b_) as well as a coefficient related to wind effects on thermal conductance for a “typical passerine” (0.26). For *T*_e_, we used daily average operative temperatures ([daily maximum *T*_e_ + daily minimum *T*_e_]/2), and for *T*_b_, we used 41°C. To estimate *u* experienced at our microclimate study locations, we used the South Dakota State Mesonet daily weather summary archives (https://climate.sdstate.edu/archive/) at the Beresford, SD, weather station, ∼35 km from our study sites. We used the daily average wind speeds for each of the days we recorded operative temperature. For open sites, we simply used the average wind speeds as *u* in our *T*_es_ calculations. For the sheltered sites, however, we needed to consider the potential reductions in wind speed due to vegetation cover. We estimated the percent reduction using wind speed data from Schneider (1985), which measured and compared wind speeds inside and outside of deciduous and coniferous shelterbelts in eastern South Dakota. We calculated the percent difference between average wind speeds within each shelterbelt type and average wind speeds outside the shelterbelts for December through February (for winter data) and July through August (for summer data) (see Table 4 in Schneider 1985). To reduce the weather station wind speeds to either the deciduous or coniferous condition, we multiplied the weather station wind speed measurements by the percent change then subtracted that value from the starting weather station wind speed. The final calculated wind speeds were then used in the Bakken (1990) equation to derive our sheltered standard operative temperature estimates.

### Statistics

#### Operative temperature

We calculated daily minimum and daily maximum *T*_e_, along with single extreme minimum and maximum *T*_e_ for each season, for each *T*_e_ thermometer. To compare the impact of open versus sheltered habitats on minimum and maximum daily *T*_e_, we used a generalized linear mixed-model (GLMM) using the R package lmer4 (Bates et al. 2015), with minimum and maximum daily *T*_e_ as the dependent variables, habitat as a fixed effect and operative temperature thermometer ID and year as random factors. We compared the full GLMM with the intercept-only model containing only the random factors by a restricted maximum likelihood (REML) approach. We conducted separate GLMM analyses for summer and winter. We visually checked residual plots and verified that residuals were normally distributed in all cases. To compare the mean daily average standard operative temperature estimates (Bakken 1990), calculated using mean minimum and maximum values ([minimum *T*_e_ + maximum *T*_e_]/2) from each *T*_e_ thermometer, for open and sheltered microclimates for winter and summer seasons we used a 2-tailed, 2 sample Student’s *t*-test or Mann–Whitney *U*-tests if parametric assumptions of normal distribution or equal variance were violated.

For summer data, we calculated the percentage of *T*_e_ recordings for each *T*_e_ thermometer that exceeded 40°C (a temperature slightly above the upper critical temperature for both horned larks and house sparrows; Kendeigh 1969; Trost 1972). These comparisons were designed to test whether birds in the 2 habitat types differed in their exposure to potential thermal stress from heat. We also calculated the percentage of winter temperature recordings less than −1.4°C for house sparrows at sheltered sites and less than −8.3°C for horned larks at open sites. These temperatures are those predicted to elicit metabolic rates of 2.5× BMR for the 2 species (Hudson and Kimzey 1966; Trost 1972), a metabolic level associated with cold range boundaries for a number of bird species (Root 1988; Buckley et al. 2018). We calculated separate values for these variables for each season and year. We analyzed the 2 years separately to determine if year-to-year differences that might influence changes in metabolic rates occurred. We compared the arcsine-square root transformed monthly mean percentages exceeding winter thresholds for each microclimate type using 1-way ANOVA to determine if monthly mean percentages differed for either year. For summer data, we compared the arcsine-square root transformed mean percentages of time points exceeding 40°C for each microclimate type using a 2-tailed Student’s *t*-test.

#### Body mass, M_sum_, and BMR

We compared mean seasonal variation in body mass (*M*_b_) before summit and BMR measurements for both species. We performed seasonal comparisons with a 2-tailed, 2 sample Student’s *t*-test, or a Mann–Whitney *U*-test if parametric assumptions of normal distribution or equal variance were violated. We calculated whole-organism (W-O) *M*_sum_ and BMR from oxygen consumption measurements using ExpeData version 2.0 software (Sable Systems, North Las Vegas, NV) and appropriate formulas from Lighton (2008). We determined *M*_sum_ using the instantaneous correction (see Bartholomew et al. 1981) with the highest running 5-min mean period of oxygen consumption over the cold exposure trial considered as *M*_sum_ (Wiersma et al. 2007; Swanson et al. 2012). For BMRs, we used the lowest 10-min running mean of oxygen consumption during the overnight trial calculated according to steady-state conditions (Lighton 2008). We corrected all metabolic rates to Standard Temperature Pressure Dry, except for the FoxBox measurements of BMR for several house sparrows, as these measurements employed mass flow rates. We used a 2-tailed Student’s *t*-test to compare mean metabolic rates between seasons for both species, or Mann–Whitney *U*-tests if parametric assumptions were violated. We also used GLMs to analyze seasonal and species-specific variation in metabolic rates, with separate GLMs for BMR and *M*_sum_. Because a principal mechanism of seasonal metabolic flexibility in birds involves changes in body composition and body mass (Swanson 2010), we conducted these GLMs both with and without body mass as a covariate. For W-O data, BMR and *M*_sum_ were the dependent variables and species, season and the season * species interaction term was independent variables. For mass-adjusted analyses, GLMs included log_10_ BMR and log_10_  *M*_sum_ as dependent variables, and species, season and the season * species interaction term as independent variables, with log_10_ body mass as a covariate.

We considered *P *<* *0.05 to represent statistical significance for all statistical tests. We conducted statistical tests other than GLMs with Sigma Stat version 3.5 (Systat Software, Inc., Point Richmond, CA). All GLM and GLMM analyses were conducted in R Version 3.6.1 (R Core Team 2019).

## Results

### Operative temperature (*T*_e_)

**Figure 1.**
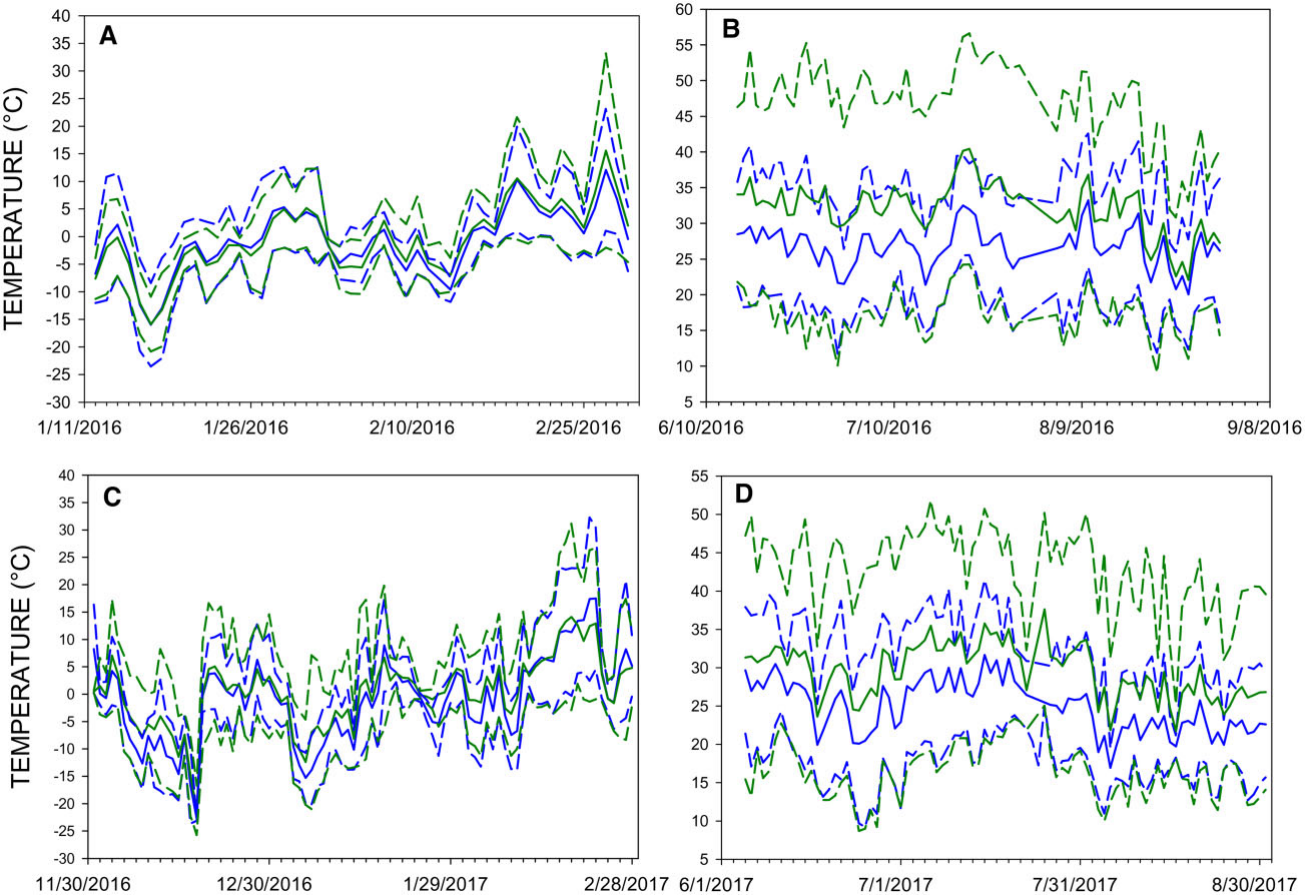
Daily average operative temperatures (*T*_e_) (solid lines; [*T*_min_* *+* T*_max_]/2) and average minimum and maximum *T*_e_ (dashed). Horned lark *T*_e_ (open sites) are in green and house sparrow *T*_e_ (sheltered sites) are in blue. Panels are: (A) Winter 2016 (January–February), (B) Summer 2016 (June–August), (C) Winter 2016–2017 (December–February), and (D) Summer 2017 (June–August).

Operative temperatures were generally more similar between the 2 microclimate types in winter (Figure 1A,C) than in summer (Figure 1B,D). The full model GLMMs with habitat included as a fixed effect consistently performed better than the intercept-only model without habitat, suggesting that habitat is an important effector of minimum and maximum daily *T*_e_ in this study (Table 1). Minimum daily *T*_e_ was slightly higher in sheltered than in open habitats (Figure 1) during both summer and winter, but the difference between habitats was weak in winter (*t*_1375_* *=* *1.589) and only slightly stronger in summer (*t*_1375_* *=* *2.436). Statistical inference from *t*-statistics of linear mixed models is complicated because mixed model parameters lack asymptotic distributions (Bates et al. 2015), but *P*-values resulting from these *t*-statistics were 0.112 in winter and 0.015 in summer. Maximum daily *T*_e_ showed much stronger between-habitat differences, with maximum *T*_e_ always higher in open than in sheltered habitats in both summer and winter (*P *<* *0.001 at both seasons). Because daily minimum *T*_e_ was lower and daily maximum *T*_e_ higher in open than in sheltered sites at both seasons (Figure 1), this signifies a greater range of operative temperatures experienced throughout the day in open than in sheltered sites.

**Table 1.** Results of GLMM analyses on minimum and maximum operative temperature (*T*_e_) as a function of habitat occupied by horned larks and house sparrows (open versus sheltered habitats, respectively)

Season	Daily *T*_e_	Model	Log-likelihood	REML	Habitat
Summer	Minimum	Intercept-only	−3,650.4	7,300.8	—
		Full	−3,647.7	7,295.4	0.982
Summer	Maximum	Intercept-only	−4,504.1	9,008.2	—
		Full	−4,495.0	8,990.4	−11.309
Winter	Minimum	Intercept-only	−3,748.6	7,497.2	—
		Full	−3,747.0	7,493.9	0.962
Winter	Maximum	Intercept-only	−4,150.3	8,300.6	—
		Full	−4,135.2	8,270.4	−6.343

Column headings are log-likelihood and REML estimates of model performance and the model estimates for the habitat variable, with the sheltered habitats as the basis for comparison. Positive and negative values for the habitat variable indicate higher or lower values, respectively, for *T*_e_ in sheltered versus open habitats.

**Figure 2.**
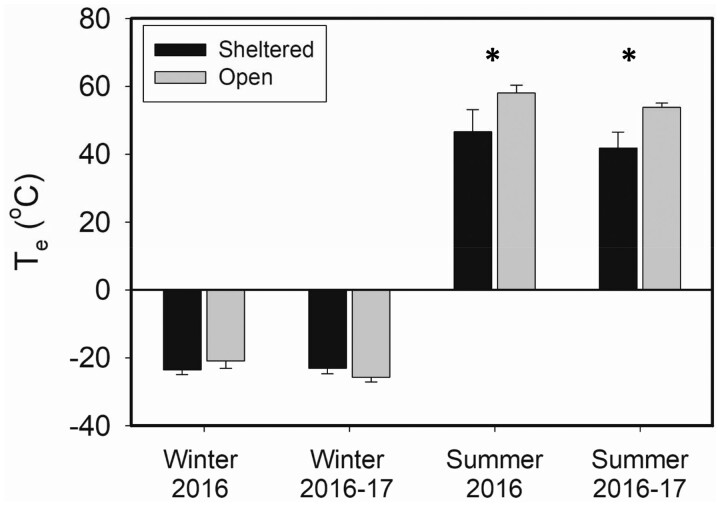
Mean extreme minimum and maximum *T*_e_ for each microclimate type during each field season. Sheltered habitats were occupied by house sparrows (Sheltered; black) and open habitats were occupied by horned larks (open; gray). *T*_e_ did not differ between years for either microhabitat, so we pooled values for comparisons. Winter *T*_e_ did not differ significantly between sites, but summer *T*_e_ was significantly hotter (asterisks) in open than in sheltered sites for both years.

Mean extreme minimum and mean extreme maximum operative temperatures for each microclimate type and for each season depict the coldest and hottest mean operative temperatures that occurred in each microclimate type for winter and summer (Figure 2). Winter mean extreme minimum operative temperatures did not differ significantly between years (Figure 2) for sheltered sites (*t*_7_* *=* *0.183, *P *=* *0.860) or open sites (*t*_7_* *=* *1.964, *P *=* *0.090), so we pooled values for the 2* *years for comparisons of open versus sheltered sites. Mean extreme minimum *T*_e_ for the 2* *years combined did not differ significantly (*t*_16_* *=* *0.503, *P *=* *0.622) between open and sheltered sites. The single coldest operative temperatures from sheltered and open sites were −27.2°C and −29.4°C, respectively. Summer mean extreme maximum operative temperatures did not differ significantly between years for either open or sheltered sites (*P *>* *0.127 for both sites). Pooled values for mean extreme maximum *T*_e_ for the 2 study years were significantly greater (*t*_18_* *=* *2.563, *P *=* *0.020) in open (55.3 ± 1.3°C) than in sheltered (44.0 ± 3.8°C) sites. Overall, the single hottest operative temperatures recorded for sheltered and open sites were 66.1°C and 61.8°C, respectively.

**Figure 3.**
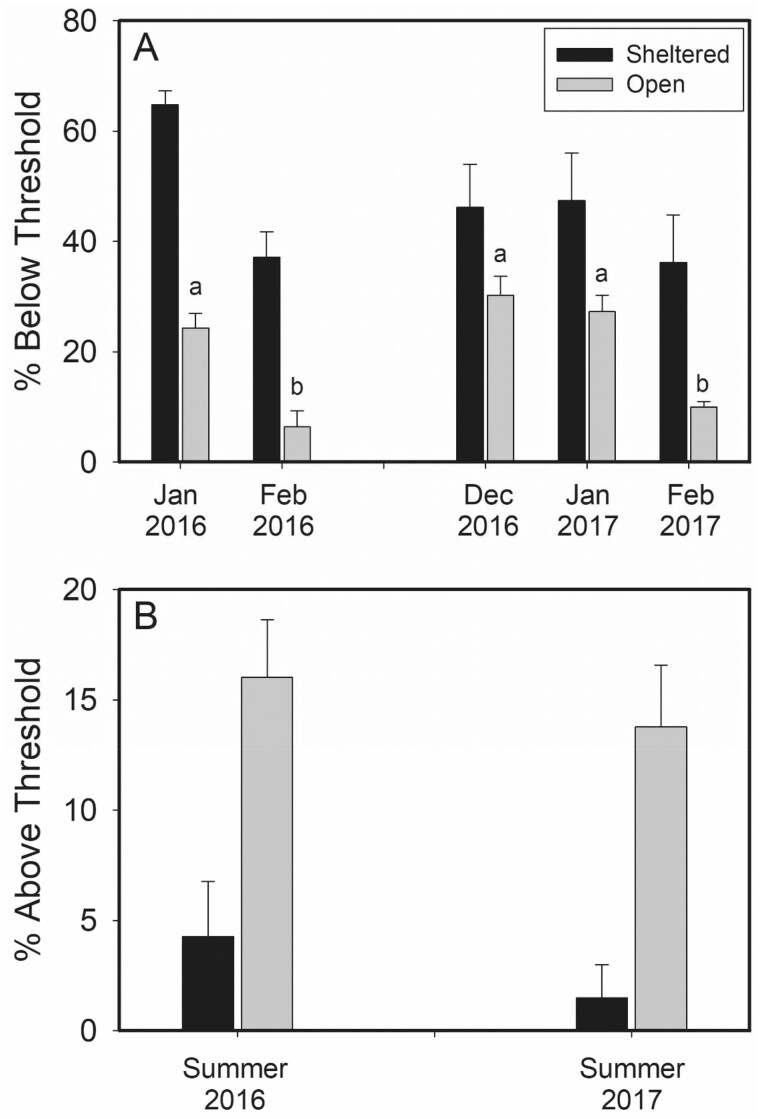
(A) Mean percentage of operative temperatures inducing an estimated metabolic rate of ≥2.5× BMR (below threshold) for horned larks in open microclimates and house sparrows in sheltered microclimates during winter. (B) Mean percentage of operative temperatures >40°C (a temperature inducing evaporative cooling) (above threshold) for horned larks in open microclimates and house sparrows in sheltered microclimates during summer. Different lower-case letters over bars denote significant differences among months.

To determine how often each species is potentially faced with thermal stress (i.e., having to increase metabolic rates for thermogenesis or evaporative cooling to maintain body temperature), we compared the percentage of operative temperatures throughout the winter season where operative temperatures fell below temperatures eliciting a metabolic rate of 2.5× BMR (−1.4°C for house sparrow, −8.3°C for horned lark; see the “Materials and Methods” section; Figure 3A) or throughout the summer season where *T*_e_ exceeded 40°C (Figure 3B). For sheltered sites, no significant differences in the percentages of time less than −1.4°C occurred among months for either year. For open sites, however, operative temperatures exceeded the threshold temperatures producing metabolic rates ≥2.5× BMR in horned larks for greater percentages of time in December/January than in February for both winters (Figure 3A). Statistics for these comparisons were: 2016, January versus February, *t*_6_* *=* *3.854, *P *=* *0.009; 2017, December versus February, *t*_9_* *=* *4.440, *P *=* *0.006; 2017, January versus February, *t*_9_* *=* *3.875, *P *=* *0.007.

During the summer of 2016, the percentage of operative temperatures >40°C for sheltered sites was marginally not significantly different from the percentage of operative temperatures at open sites above 40°C (*U*_5_* *=* *0.0, *P *=* *0.057; Figure 3B). Open sites had significantly higher percentages of *T*_e_ above 40°C (*U*_9_* *=* *2.0, *P *=* *0.017) than sheltered sites during the 2017 summer (Figure 3B). Thus, horned larks at open sites generally experienced more potentially stressful *T*_e_ during the summer than house sparrows at sheltered sites.

#### Standard operative temperature (T_es_) estimates

Including convective effects on microclimates by estimating *T*_es_ revealed contrasting results from *T*_e_ measurements in winter. In both winters, open microclimates exhibited significantly colder daily average *T*_es_ than for both deciduous and coniferous sheltered microclimates (Table 2). Wind chill markedly cooled microclimate temperatures for both open and sheltered sites during the winter, but especially for open habitats. For the summer seasons, estimated *T*_es_ displayed similar differences between open and sheltered microclimates as did *T*_e_ measurements (Tables 1 and 2). Mean daily average *T*_es_ for open sites was significantly hotter than *T*_es_ for both deciduous and coniferous sites for both years (Table 2). Convection cooled summer daily average microclimate temperatures at both open and sheltered microclimates but did not fully compensate for the higher radiative heat loads at open sites.

**Table 2.** Estimated standard operative temperatures (*T*_es_ (± *SE*) using wind speeds estimated for deciduous (D; sheltered), coniferous (C; sheltered), and outside (O; open) habitats and body temperatures (*T*_b_) of 41°C

Season	Open	Deciduous	Coniferous	O versus D statistic	O versus D *P*-value	O versus C statistic	O versus C *P*-value
Winter 2016	−22.6 ± 1.5	−15.1 ± 1.2	−16.7 ± 1.3	*t*_96_ = 3.866	<0.001	*t*_96_ = 2.947	0.004
Winter 2016–17	−19.1 ± 1.1	−13.8 ± 1.1	−15.5 ± 1.1	*t*_178_ = 3.361	<0.001	*t*_178_ = 2.286	0.023
Summer 2016	28.3 ± 0.6	22.7 ± 0.4	22.2 ± 0.4	*U*_143_ = 4343	<0.001	*U*_143_ = 4242	<0.001
Summer 2017	25.1 ± 0.5	21.3 ± 0.5	20.8 ± 0.5	*t*_171_ = 5.232	<0.001	*t*_171_ = 5.846	<0.001

#### Seasonal variation in body mass and metabolic rates

**Figure 4.**
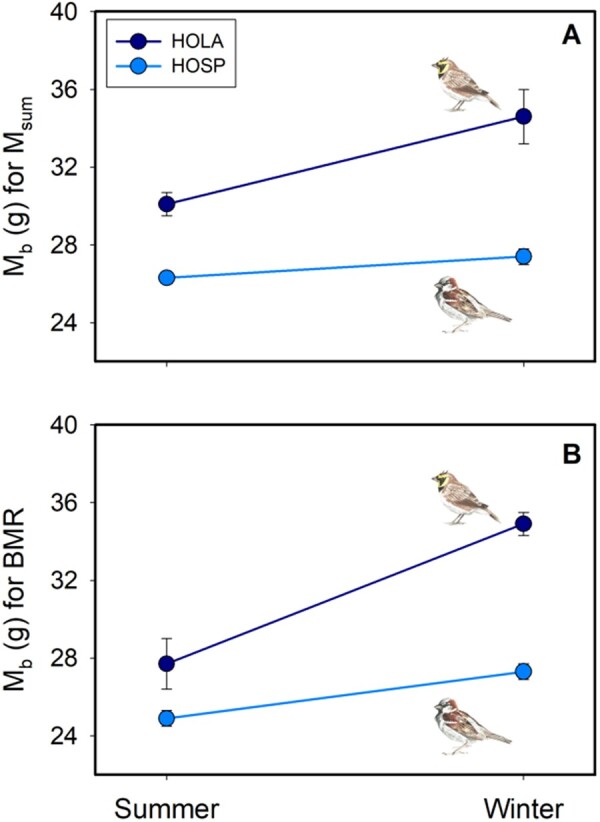
Mean (± *SE*) body mass before (A) summit (*M*_sum_) and (B) BMR measurements for horned larks (HOLA, navy) and house sparrows (HOSP, blue) during summer and winter.

Seasonal variation in *M*_b_ measured prior to *M*_sum_ measurements varied between house sparrows and horned larks (Figure 4A). Mean winter *M*_b_ in house sparrows was significantly (4.6%, *t*_29_* *=* *2.393, *P *=* *0.023) greater than mean summer *M*_b_. Horned larks did not show a significant (*U*_9_* *=* *24, *P *=* *0.073) seasonal change in mean *M*_b_ prior to *M*_sum_ measurements, despite a 15% increase in winter relative to summer (Figure 4A). Mean winter *M*_b_ before BMR measurement was significantly (horned larks: *U_5_ *=* *12.0, *P *=* *0.003; house sparrows: *t*_30_* *=* *3.692, *P *<* *0.001) greater than mean summer *M*_b_ for both horned larks (26%) and house sparrows (9.6%) (Figure 4B).

**Figure 5.**
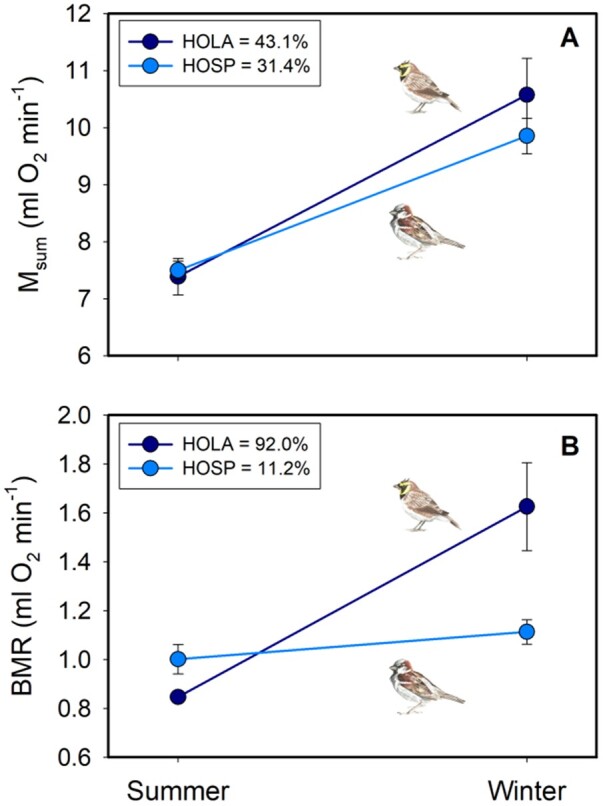
Mean (± *SE*) summit (*M*_sum_; A) and BMR (B) for horned larks (HOLA, navy) and house sparrows (HOSP, blue) during summer and winter. Percent winter increases are provided for each species and metabolic rate in the legend.

Both house sparrows and horned larks experienced seasonal changes in metabolic capacity (Figure 5). Winter mean *M*_sum_ for horned larks was significantly greater (43.1%; *t*_9_* *=* *3.532, *P *=* *0.006) than the mean summer *M*_sum_ (Figure 5A). Similarly, house sparrow winter *M*_sum_ was significantly greater (31.4%; *U*_29_* *=* *220.0; *P *<* *0.001) than summer *M*_sum_ (Figure 5A). Horned lark and house sparrow BMR differed in their responses to the changing seasons (Figure 5B). There was no significant seasonal change in BMR for house sparrows (*t*_28_* *=* *1.423, *P *=* *0.166). Horned lark BMR did vary seasonally with mean winter rates significantly (*t*_5_* *=* *5.315, *P *=* *0.003) exceeding mean summer rates by 92%.

Season was a significant predictor of both *M*_sum_ and BMR for W-O GLMs, but BMR also showed a significant season * species interaction term, with horned larks showing greater winter increases in BMR than house sparrows (Table 3). The season * species interaction term showed the same trend for *M*_sum_, but was not significant, and species was not a significant effector of either *M*_sum_ or BMR for W-O GLMs. The mass-adjusted GLM results indicated significant positive effects of body mass on both *M*_sum_ and BMR (Table 3). After correcting for body mass effects on metabolic rates, the main effect of season was a significant effector of *M*_sum_, with higher mass-adjusted *M*_sum_ in winter than in summer (Table 3). Species and the species * season interactions were not significant effectors of mass-adjusted *M*_sum_ (Table 3). Species was a significant effector of mass-adjusted BMR, with house sparrow mass-adjusted BMR higher than for horned larks (Table 3). Season and the species * season interaction were not significant effectors of mass-adjusted BMR (Table 3).

**Table 3.** Results of GLMs for metabolic rates on W-O and mass-adjusted (log *M*_sum_ and log BMR) bases as functions of season, species, and species * season interaction variables

Metabolic rate	*df*	*R* ^2^	Variable	Estimate	*SE*	*t*-value	*P*-value
W-O *M*_sum_	38	0.552	**Season**	3.187	0.758	4.207	**< 0.001**
			Species	0.110	0.698	0.158	0.875
			Spp * Season	−0.832	0.879	0.947	0.350
W-O BMR	33	0.463	**Season**	0.779	0.171	4.550	**< 0.001**
			Species	0.153	0.147	1.067	0.293
			Spp * Season	−0.666	0.190	3.506	**0.001**
log *M*_sum_	37	0.681	**log *M*_b_**	0.734	0.272	2.698	**0.010**
			**Season**	0.252	0.078	3.234	**0.003**
			Species	0.118	0.074	1.600	0.118
			Spp * Season	−0.019	0.083	0.230	0.820
log BMR	32	0.553	**log *M*_b_**	1.919	0.553	3.473	**0.002**
			Season	0.192	0.190	1.007	0.321
			**Species**	0.344	0.132	2.617	**0.013**
			Spp * Season	−0.251	0.175	1.438	0.160

For mass-adjusted analyses, log body mass (*M*_b_) was included as a covariate. Variables with a significant effect on metabolic rates are highlighted in bold.

## Discussion

Our results documenting generally greater seasonal variation in metabolic rates for horned larks than for house sparrows are consistent with the predictions of the CVH when extended to the level of sympatric species inhabiting different microclimates. The open microclimates of horned larks produced greater seasonal variation in operative and estimated standard operative temperatures, which were associated with larger seasonal differences in *M*_sum_ and BMR than for house sparrows, whose sheltered microclimates exhibited less daily and seasonal thermal variation. Open habitats produced colder winter and hotter summer microclimate temperatures than the sheltered habitats, suggesting greater metabolic demands for horned larks for thermogenesis in winter and for reductions in heat production in summer. These results suggest that extrapolation of the CVH to sympatric species occupying microclimates differing in temperature variability might be appropriate.

Both horned larks and house sparrows demonstrated seasonal phenotypic flexibility in metabolic capacity to match the changing energetic demands of the environment (Figure 5). The magnitude of the seasonal variation in *M*_sum_ tracked differences in the metabolic costs induced by the microclimates to which each species was exposed. Horned larks showed an increase in *M*_sum_ of 43.1% from summer to winter compared with only 31.4% for house sparrows, which suggests that the more seasonally variable environmental temperatures of horned lark microclimates (Figures 1 and 2) produce greater seasonal variation in metabolic capacity. The lower estimated winter *T*_es_ that horned larks experience in open microclimates would be expected to generate higher *M*_sum_ than for house sparrows from relatively milder, sheltered microclimates. Winter *M*_sum_ for horned larks was 7.6% greater than winter *M*_sum_ for house sparrows, but this increase can largely be explained by the larger body size of horned larks rather than by variation in winter environmental temperatures. In addition, species was not a significant predictor of *M*_sum_ for either W-O or mass-adjusted analyses, suggesting that species differences in *M*_sum_ were not marked. Winter *M*_sum_ exceeded interspecific allometric predictions (Swanson and Liknes 2006) by 14.2% and 21.6% for horned larks and house sparrows, respectively, suggesting that both species generate high *M*_sum_ in the cold winter climates of South Dakota.

Another potential factor that may have influenced the magnitude of the seasonal variation in horned lark *M*_sum_ is the failure to elicit hypothermia in some larks. Most of the winter horned lark individuals exhibited strong cold endurance by lasting either nearly 3 h (the maximum time for which we collected *M*_sum_ measurements, *n = *4) or failing to become hypothermic before the 3-h limit (*n = *3), whereas all house sparrows became hypothermic before 3 h of cold exposure. During *M*_sum_ measurements, larks experienced temperatures down to −19°C in helox (the lowest temperature that our equipment could reach), and some birds did not become hypothermic, so winter *M*_sum_ values for larks are conservative and may not represent true maximum rates. Nevertheless, rates of oxygen consumption over the last hour of *M*_sum_ measurements for larks that did not become hypothermic were relatively stable, suggesting that rates had reached close to peak levels. In addition, Swanson et al. (1996) suggested that static temperatures in helox that produced hypothermia in 50% of individuals produced similar mean *M*_sum_ to sliding exposure to declining temperatures, and 57.1% of horned larks did become hypothermic under the cold exposure conditions in this study. Thus, although we cannot rule out that mean *M*_sum_ in horned larks may have been slightly higher if we had elicited hypothermia in all individuals, the mean winter *M*_sum_ values that we report for horned larks likely approach true *M*_sum_ closely.

A larger winter body mass than house sparrows (Figure 4) may enable horned larks to sustain higher metabolic capacity, especially if the winter increase in *M*_b_ can be attributed to enlargement of thermogenic muscles which have the potential to produce more heat through shivering (Swanson 2010; Milbergue et al. 2018). Seasonal increases in cellular metabolic capacity (Milbergue et al. 2018), through increases in activity of key aerobic enzymes such as citrate synthase, cytochrome c oxidase, and β-hydroxyacyl CoA-dehydrogenase in these muscles, can also contribute to elevated *M*_sum_ and improved cold endurance (Marsh and Dawson 1989; Liknes and Swanson 2011a, 2011b; Li et al. 2020). Thermal conductance, calculated from the interspecific allometric equation of Schleucher and Withers (2001), is also predicted to be 11.3% lower in horned larks than in house sparrows because of their larger *M*_b_. The combination of a larger *M*_b_, lower thermal conductance and a high winter *M*_sum_ may enable horned larks to cope with cold conditions better than house sparrows, consistent with the high tolerance to cold of horned larks in this study. This conclusion is consistent with the lower percentage of time that horned larks exceeded *T*_e_ thresholds for elevating metabolic rates to ≥2.5× BMR relative to house sparrows in this study.

In summer, horned larks and house sparrows had similar *M*_sum_ (Figure 5) despite the hotter daily average and maximum *T*_e_ at open sites (Figure 1), as well as a greater occurrence and duration of potentially thermally stressful conditions (Figure 3). These factors should induce a greater reduction in metabolic capacity and possibly a lower *M*_sum_ in summer for larks than for sparrows. The similar summer *M*_sum_ for both species can be at least partially explained by *M*_b_ differences between species because summer larks were 13.9% larger than summer house sparrows but had 1.5% lower *M*_sum_. Nevertheless, species was not a significant effector of *M*_sum_ for either W-O or mass-adjusted analyses (Table 3), suggesting only minor differences in *M*_sum_ between horned larks and house sparrows. In addition, summer *M*_sum_ did not exceed allometric predictions (Swanson and Liknes 2006) by as great a magnitude as winter *M*_sum_ for either horned larks (2.0%) or house sparrows (14.1%).

The results in this study are generally consistent with other studies on house sparrows regarding seasonal variation in *M*_sum_. Swanson and Liknes (2006) documented an 11% increase from summer to winter for *M*_sum_ for this same population of house sparrows. This seasonal *M*_sum_ variation is smaller than that observed in this study (31.4%), although winter *M*_sum_ values in the 2 studies were within 5.5% of each other. The difference in the magnitude of seasonal change in *M*_sum_ between these 2 studies is driven primarily by the 12.3% higher summer *M*_sum_ recorded by Swanson and Liknes (2006). Other studies of seasonal variation in *M*_sum_ for house sparrows, however, showed winter increases similar to those in this study (Hart 1962; Arens and Cooper 2005). House sparrows from Ontario experienced a 43% increase in winter *M*_sum_ whereas sparrows from Wisconsin exhibited a 31% increase (Hart 1962; Arens and Cooper 2005).

Significant seasonal variation in BMR was detected in this study only for horned larks, with a 92% increase from summer to winter, whereas house sparrows showed a non-significant 11% winter increase (Figure 5). In addition, the season * species interaction was a significant predictor of W-O BMR, with greater seasonal flexibility for horned larks than for house sparrows. This significant effect of the season * species interaction disappeared for mass-adjusted BMR analyses (Table 3), suggesting that mechanistic underpinnings of the greater seasonal variation in BMR in larks involved increases in *M*_b_ and changes in body composition in response to the more marked seasonal changes in microclimate temperatures. Higher winter and lower summer BMR in horned larks allow support of greater thermogenic capacity in colder winter microclimates and reduced metabolic heat production in hotter summer microclimates. These changes should benefit tolerance by larks to colder winter and higher summer microclimate temperatures in their open, exposed habitats (Beason 1995).

The absence of significant seasonal variation in BMR for house sparrows in this study is not consistent with other studies. For example, house sparrows from Wisconsin (Arens and Cooper 2005) and South Africa (Nzama et al. 2010) showed 64% and 220% increases in BMR, respectively, from summer to winter. For South African sparrows, variation in daily temperatures and unpredictable winter conditions explained most of the seasonal variation (Nzama et al. 2010) observed in sparrow BMR, which could help explain the difference from results in this study. In addition, the reduction in metabolic costs afforded by the protective and insulative qualities of sheltered microclimates available to free-living birds might also contribute to differences between studies, as South African sparrows were housed in outdoor aviaries before experimentation, which could limit access to the full range of microclimates available under natural conditions. Similar effects on acclimation capacity of BMR between captive and wild populations have been previously documented for other bird species (Maldonado et al. 2009). However, Wisconsin sparrows also showed much greater seasonal BMR variation than South Dakota birds in this study and climatic conditions in Wisconsin are similar to those in southeastern South Dakota (Olson et al. 2010), so reasons for the reduced seasonal BMR variation between these populations are unknown.

Both species in this study showed capacity for flexibility of *M*_sum_ and/or BMR, similar to other temperate-zone and subtropical bird species (Swanson 2010; McKechnie et al. 2015). The ability to adjust metabolic rate to environmental temperatures (Nilsson and Nilsson 2016; Petit et al. 2017; Latimer et al. 2018) and use available microclimates to reduce thermally stressful conditions (e.g., Buttemer 1985; Cooper 1999) can increase chances of survival. Examination of actual microclimate conditions available to birds can provide insight into the metabolic demands they impose on species. With the threat of increasing occurrences of extreme weather due to climate change (Crick 2004), understanding the relationship between microclimate heterogeneity and metabolic flexibility can assist in the assessment of species vulnerability to dramatic changes in environmental temperatures. Further study into this relationship can also have potential conservation implications, because habitat loss may reduce available microclimates, which in turn, have energetic costs associated with the absence of the full range of microclimate options (Maldonado et al. 2009).

Future studies using standard operative temperature models (Bakken et al. 1981; Dzialowski 2005) to more fully incorporate convective cooling into comparisons of thermal conditions would be beneficial to more completely understand the relationships between microclimate variability and metabolic flexibility in birds. In addition, because this was a study involving only one species from each habitat under study, we cannot rule out that other factors which could differ between the 2 species might contribute to the differences detected in this study. Thus, we can only say that our results did not falsify the predictions of the CVH applied to sympatric species occupying different microclimates and cannot directly confirm that microclimate temperatures influence metabolic flexibility more generally for small birds (Garland and Adolph 1994). As such, future research performed on a broader range of species is required to establish whether this relationship applies generally and to elucidate the finer details of this pattern and the other potential factors that might play roles in regulating metabolic flexibility.

## Author Contributions

P.O. and D.L.S. designed the study, collected and analyzed microclimate and metabolic rate data, and wrote and edited the manuscript.
